# Intestinal ultrasound in acute severe ulcerative colitis treated with intravenous cyclosporine: case series

**DOI:** 10.1093/crocol/otag037

**Published:** 2026-04-29

**Authors:** Yusuke Miyatani, Joëlle St-Pierre, Jeremy Klein, David T Rubin, Noa Krugliak Cleveland

**Affiliations:** University of Chicago Medicine Inflammatory Bowel Disease Center, Department of Medicine, Chicago, IL, United States; University of Chicago Medicine Inflammatory Bowel Disease Center, Department of Medicine, Chicago, IL, United States; University of Chicago Medicine Inflammatory Bowel Disease Center, Department of Medicine, Chicago, IL, United States; University of Chicago Medicine Inflammatory Bowel Disease Center, Department of Medicine, Chicago, IL, United States; University of Chicago Medicine Inflammatory Bowel Disease Center, Department of Medicine, Chicago, IL, United States

**Keywords:** cyclosporine, acute severe ulcerative colitis, intestinal ultrasound

## Abstract

**Background:**

Intestinal ultrasound (IUS) has been studied to assess therapeutic response in patients with acute severe ulcerative colitis (ASUC). However, there is no data on sonographic changes occurring in ASUC receiving intravenous (IV) cyclosporine. This case series aims to describe the sonographic changes occurring in hospitalized patients with ASUC receiving IV cyclosporine as salvage therapy.

**Methods:**

This is a retrospective observational study in a single tertiary care center from February 2023 to May 2024. Serial sonographic findings of patients with severe UC treated with IV cyclosporine were collected. Sonographic changes associated with clinical outcomes were analyzed. Outcomes included clinical response (Simple Clinical Colitis Activity Index decrease of ≤3 at day 3 with discharge on oral cyclosporine) and colectomy.

**Results:**

Nine patients received IV cyclosporine with IUS monitoring; 67% failed ≥2 advanced therapies. Four responded to cyclosporine, four underwent colectomy, and one switched to upadacitinib with a subsequent response during the same admission. Clinical responders had a median 31.8% (IQR 26.3-51.2) reduction in bowel wall thickness (BWT) and 1-scale reduction in modified Limberg score, whereas non-responders demonstrated 7.2% (IQR 54.8-9.0) reduction in BWT and no change in modified Limberg score. The median time to clinical response was 2.5 days (IQR 1.8-2.6). Baseline BWT was similar between responders and non-responders: 5.2 mm (IQR 3.5-6.5) versus 5.2 mm (3.8-6.7).

**Conclusions:**

A median 31.8% BWT reduction was observed by day 3 among responders, highlighting early sonographic changes associated with response to IV cyclosporine therapy in refractory ASUC.

## Graphical Abstract

**Graphical Abstract:**
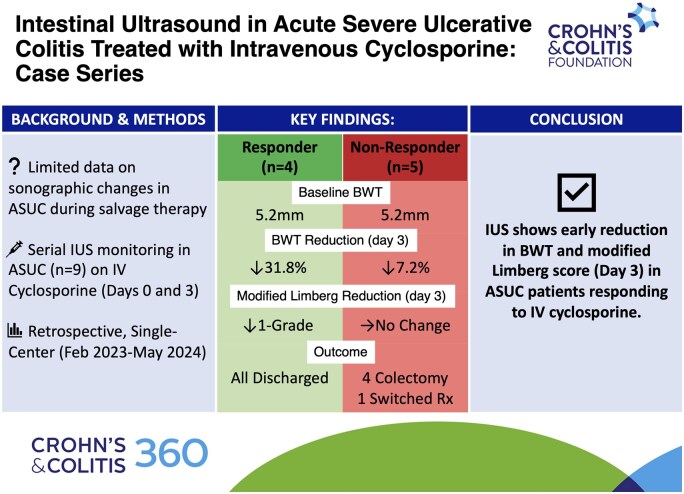

## Introduction

Ulcerative colitis (UC) is a chronic inflammatory condition that mainly affects the colon.^1^ Approximately 25% of patients with UC experience acute severe UC (ASUC),^2^ which has been traditionally defined by the Truelove and Witts criteria as frequent bloody bowel movements (≥6/day) accompanied by systemic toxicity.^3^ Up to 15％ may eventually require a colectomy.^4^ One in 4 patients with ASUC may not respond to IV corticosteroids and may ultimately require salvage therapies, which include infliximab, Janus kinase inhibitors, or IV cyclosporine.^5–8^ Cyclosporine, a calcineurin inhibitor, is one of the salvage therapies used for ASUC.^9^ A pivotal randomized controlled trial showed 82% of patients with steroid-refractory severe UC responded to cyclosporine.^10^ Yet, real-world long-term data that included patients with prior failure of advanced therapies showed lower success, and more than 50% of the patients subsequently required colectomy after cyclosporine treatment.^11^^,^^12^ Although cyclosporine is part of the salvage therapy armamentarium for ASUC, there is still a significant risk of colectomy, especially for patients who have failed multiple prior advanced therapies.^11^

Effective monitoring of response to salvage therapy is crucial in managing ASUC. A timely assessment of therapeutic response to salvage therapy helps prevent surgical delay, resulting in better clinical outcomes, including mortality and post-surgical complications.^13–15^ Current monitoring strategies rely on patient-reported symptoms and biomarkers such as C-reactive protein (CRP), which do not always accurately reflect the degree of mucosal inflammation.^16^ While colonoscopy remains the gold standard for assessing mucosal inflammation, it is time-consuming, invasive, and requires bowel preparation for patients who are already symptomatic. This underscores the urgent need for more reliable, non-invasive monitoring tools that can provide accurate, real-time assessments of disease activity in patients treated with salvage therapy.

Intestinal ultrasound (IUS) is emerging as a valuable non-invasive tool for real-time monitoring in ASUC. Ilvemark et al. reported that a reduction in bowel wall thickness (BWT) of more than 20% by 48 ± 24 h in patients with ASUC receiving intravenous (IV) corticosteroids was predictive of response within a week.^17^ Additionally, a recent study of patients with ASUC showed that baseline transmural assessment with IUS had a higher predictive value for future colectomy compared to baseline endoscopic assessment.^18^ To date, there are no studies that describe the timeline of sonographic changes for patients suffering from ASUC treated with salvage IV cyclosporine. Our case series aims to describe early sonographic changes in hospitalized patients with ASUC treated with IV cyclosporine and their association with clinical response and non-response.

## Materials and methods

We conducted a retrospective observational study in a single tertiary IBD center from February 2023 to May 2024. Adult patients (≥18 years) with severe UC, requiring hospitalization for UC disease relapse, and monitored with serial IUS before and after starting IV cyclosporine were included. Patients younger than 18 years and those with a prior history of colectomy were excluded. Clinical information and IUS parameters were collected, including BWT, modified Limberg score (a semi-quantitative Doppler-based scale ranging from 0 [absent] to 3 [marked] for assessment of bowel wall vascularity, modified from the original Limberg score by excluding BWT)^19^ on the day of cyclosporine initiation and at follow-up IUS after cyclosporine initiation. Other sonographic data collected, including mesenteric fat hypertrophy and bowel wall stratification, were also collected in a binary fashion (present or absent). Follow-up IUS assessment data were collected at day 3 (or the closest date of IUS examination to day 3) from IV cyclosporine treatment initiation. In our practice, IUS is routinely repeated during hospitalization after initiation of salvage therapy within an approximately 3-day interval. This is based on prior evidence demonstrating sonographic response to IV corticosteroids within 72 hours,^17^ and the treatment-refractory nature of our cohort. The Milan Ultrasound Criteria (MUC) was calculated for each IUS exam, with the formula: MUC = 1.4 × bowel wall thickness [mm] + 2 × bowel wall flow, where bowel wall flow [BWF] = 1 if present or BWF = 0 if absent.^20^

The primary outcome of interest was to describe changes in IUS parameters in the clinical response and non-response groups to IV cyclosporine. Clinical response was defined as a decrease in Simple Clinical Colitis Activity Index (SCCAI) of ≥3 at day 3 following initiation of IV cyclosporine and discharge on oral cyclosporine. Changes in IUS parameters of the colectomy-free and colectomy groups during index hospitalization were also described. All IUS scans were performed by experienced gastroenterologists certified by the Intestinal Bowel Ultrasound Group criteria and with greater than 3 years of experience performing IUS. IUS was performed using the Samsung HM70 EVO machine, with linear [2-9 MHz] and convex probes [1-7 MHz]. Numerical values are shown as the median with interquartile range (IQR) and the absolute value with a percentage. Changes in IUS parameters between responder vs non-responder and non-colectomy vs colectomy were described without statistical analysis due to the small sample size.

### Ethical considerations

This study involving human participants was in accordance with the ethical standards of the institutional and national research committee and with the 1964 Helsinki Declaration and its later amendments or comparable ethical standards, with approval from the Institutional Review Board at the University of Chicago.

## Results

Nine patients with severe UC met the inclusion criteria. Baseline clinical and sonographic characteristics at the time of cyclosporine initiation are summarized in Table 1. The median age was 35 (IQR 30-42); eight (88.9%) had pancolitis, six (66.7%) had previous exposure to multiple advanced therapies, and eight (88.9%) had previous exposure to infliximab. The initial median SCCAI was 9 (IQR 6-11), and the majority (8/9) had a Mayo endoscopic subscore of 3. All patients had colonic biopsies negative for cytomegalovirus. One patient was positive for *Clostridioides difficile* detected by stool polymerase chain reaction (PCR) and treated with oral vancomycin along with IV cyclosporine. Per our standard management protocol, all patients received intravenous methylprednisolone 40 mg daily, prior to cyclosporine initiation, and did not have a clinical response at least 3 days after initiating methylprednisolone. All patients received IV cyclosporine at an initial dose of 2 mg/kg/d with dose adjustments to achieve target levels of 300-400 ng/mL. Upon confirmation of clinical response, IV cyclosporine was transitioned to oral cyclosporine and was continued for up to three months as a bridge to alternative maintenance therapy. Sonographic findings in the sigmoid colon were collected; the baseline sonographic findings at the time of cyclosporine initiation of the study population comprised a median BWT in the sigmoid colon of 5.2 mm (IQR 3.7-6.5 mm), a median modified Limberg score of 2 (IQR 1-2.5), and a median MUC score of 9.3 (IQR 7.4-11.1). A follow-up IUS was conducted at a median time of 3 (IQR 3-4) days after cyclosporine start.

**Table 1:** Baseline patient characteristics at the time of cyclosporine initiation.

		**Cyclosporine response** ^a^	
Variables	All (*N* = 9)	Responder (*N* = 4)	Non-responder (*N* = 5)
**Age (years), median (IQR)**	35 (30-42)	39.5 (35.8-50.8)	34 (23.5-38.5)
**Female sex**	4	1	3
**Disease duration (years), median (IQR)**	12 (1-15.5)	15.5 (4.3-22.3)	11 (0.5-12.5)
**Disease extent**			
**Pancolitis**	8	3	5
**Left-sided**	1	1	0
**Number of advanced therapies, median (IQR)**			
** 0**	1	0	1
** 1**	2	1	1
** ≥2**	6	3	3
**Concomitant intravenous steroid**	9	4	5
**IFX salvage therapy prior to cyclosporine**	2	1	1
***C. difficile* infection**	1	1	0
**Cytomegalovirus**	0	0	0
**SCCAI, median (IQR)**	9 (6-10.5)	7 (6-8.8)	11 (5.5-11.5)
**Hemoglobin (g/dL), median (IQR)**	10.6 (8.7-13.2)	10.8 (8.8-13.7)	10.6 (8.6-13.2)
**Albumin (g/dL), median (IQR)**	2.6 (2.2-3.4)	3.2 (2.7-3.6)	2.2 (2-2.9)
**C-reactive protein (mg/L), median (IQR)**	17 (11.5-71.5)	17 (6.5-20.8)	14 (11.5-133.5)
**Mayo Endoscopic Subscore**			
** 3**	8	4	4
** 2**	1	0	1
**BWT (mm), median (IQR)**	5.2 (3.7-6.5)	5.2 (3.5-6.5)	5.2 (3.8-6.7)
**Modified Limberg score**			
** 3**	2	0	2
** 2**	4	2	2
** 1**	3	2	1
**MUC score, median (IQR)**	9.3 (7.4-11.1)	9.2 (6.9-11.1)	9.3 (7.8-11.3)
**Loss of bowel wall stratification**	6	2	4
**Mesenteric fat hypertrophy**	7	3	4

a Response to cyclosporine is defined as a decrease in SCCAI of ≥3 at day 3 and discharge with cyclosporine. Data are presented as median (interquartile range). Abbreviations: IQR, interquartile range; SCCAI, Simple Clinical Colitis Activity Index; BWT, bowel wall thickness; MUC, Milan ultrasound criteria = 1.4 × BWT (mm) + 2 × bowel wall flow [present = 1, absent = 0].

**Figure 1:**
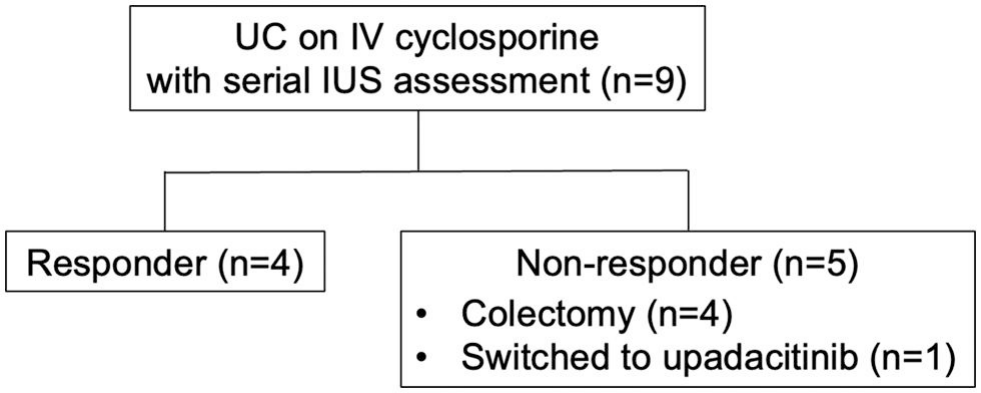
Diagram of patient inclusion and outcomes. A total of nine patients were included, and of those, four had a clinical response to cyclosporine. Five patients did not respond, and four of those had a colectomy. Abbreviations: IUS, intestinal ultrasound; IV, intravenous; UC, ulcerative colitis.

Four patients had a clinical response to IV cyclosporine with a median reduction of SCCAI of 4.5 (IQR 4-5.3) and were discharged on oral cyclosporine. Of the five non-responders, four patients underwent colectomy. (Figure 1) All colectomies were performed during the same admission at a median duration from cyclosporine start of 14 days (IQR 9.3-18.8 days). One of the five non-responders was subsequently treated with upadacitinib as salvage therapy during the same admission and demonstrated clinical response.

**Figure 2:**
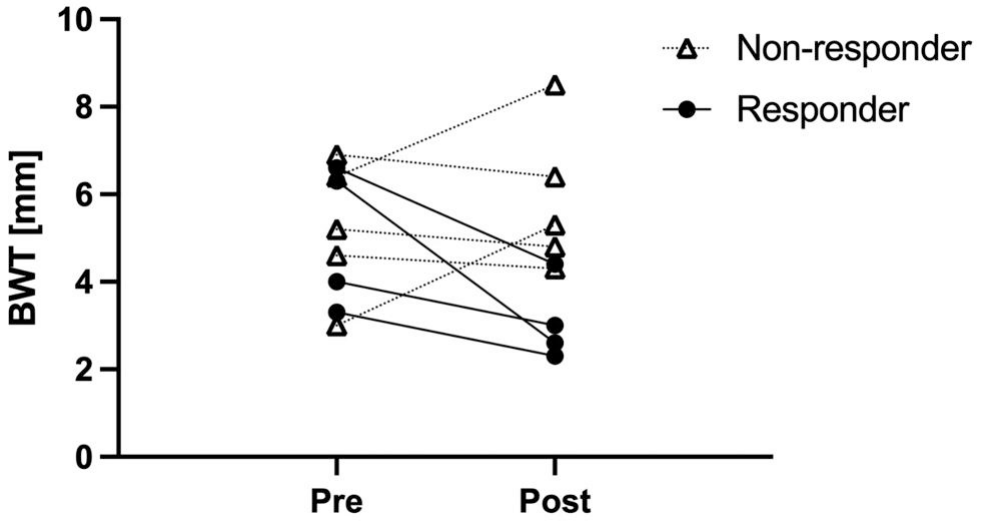
Changes in BWT at pre- and post-IV cyclosporine in responders and non-responders. Abbreviations: BWT, bowel wall thickness; IV, intravenous.

**Figure 3:**
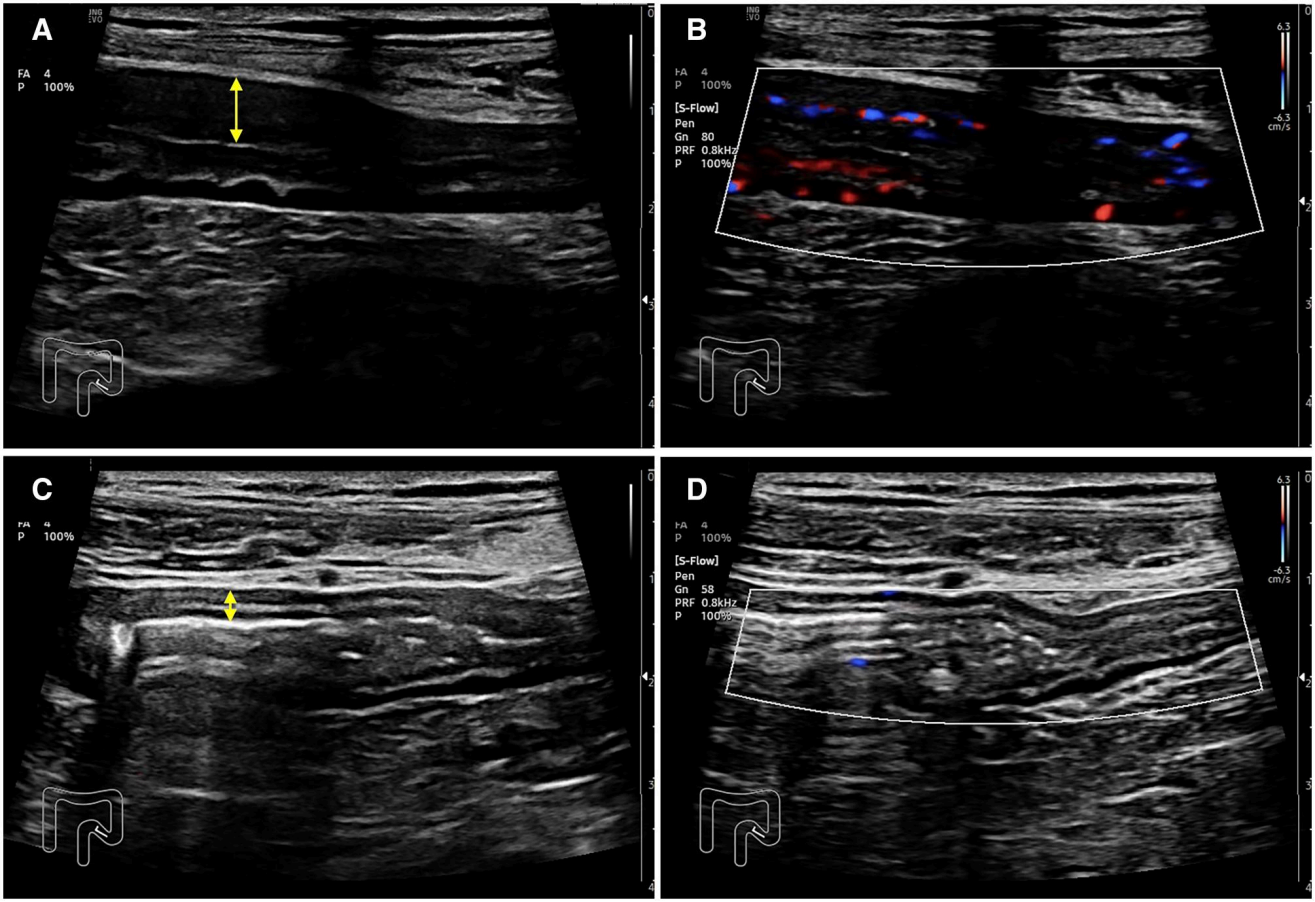
Intestinal ultrasound images demonstrating a longitudinal view of a sigmoid colon of a case who responded to intravenous (IV) cyclosporine. Before IV cyclosporine: B-mode image with increased BWT 6.2 mm (double-headed arrows) (A) and color Doppler signal with modified Limberg score 2 (B). Day 3 of IV cyclosporine: B-mode image with increased bowel wall thickness of 2.6 mm (double-headed arrows) (C) and color Doppler signal with modified Limberg score 0 (D). IV, intravenous; BWT, bowel wall thickness.

Patients with a response to cyclosporine had a median reduction in BWT of 31.8% (IQR 26.3-51.2) as compared to 7.2% (IQR −54.8 to 9.0) in non-responders (Figure 2). Responders showed a reduction in modified Limberg score by 1 point, whereas non-responders had a change of 0 (IQR −1 to 1) in modified Limberg score. Responders showed a median reduction of MUC score of 3.24 (IQR 1.8-6.2), whereas non-responders demonstrated a reduction of MUC score of 0.42 (IQR −2.6 to 0.6). Representative IUS images from a patient who responded to cyclosporine are shown in Figure 3. Patients who avoided colectomy showed a 30.3% (IQR −25.9 to 45.2%) reduction of BWT and a 1 scale reduction in the modified Limberg score with a median reduction of the MUC score of 3.1 (IQR −0.4 to 5.3) whereas patients who needed colectomy showed a 7.5% (IQR −22.8 to 9.7%) reduction of BWT and a change of 0 (IQR −0.75 to 1.5) in the modified Limberg score with a medium reduction of the MUC score of 0.5 (IQR −2.1 to 0.7). The difference in baseline BWT in each outcome was only observed between the non-colectomy and colectomy groups (Table 2). In the non-responder who subsequently responded to upadacitinib and avoided colectomy, IUS performed 3 days after initiation of cyclosporine demonstrated worsening sonographic findings, with an increase in BWT from 3.0 to 5.3 mm and an increase in the modified Limberg from 2 to 3.

**Table 2:** Changes of baseline parameters with sonographic findings after intravenous cyclosporine treatment at intestinal ultrasound follow-up.

	Cyclosporine response		Colectomy	
**Variables**	**Responder (*N* = 4)**	**Non-responder (*N* = 5)**	**Non-colectomy (*N* = 5)**	**Colectomy (*N* = 4)**
**ΔSCCAI**	4.5 (4-5.8)	2 (−0.5 to 3)	4 (1-5.5)	2 (1.3-3.5)
**ΔHemoglobin (g/dL)**	0.7 (0.2-1.5)	−0.6 (−0.75 to 0)	0.3 (0.2-1.3)	−0.7 (−0.8 to 0.3)
**ΔAlbumin (g/dL)**	0.15 (−0.08 to 0.4)	0 (−0.2 to 0.1)	0.1 (−0.05 to 0.35)	−0.05 (−0.2 to 0.08)
**ΔC-reactive protein (mg/L)**	6 (−9.8 to 13.5)	8 (−1.5 to 72.5)	0 (−0.7 to 3.0)	0.4 (−1.5 to 0.5)
**Baseline BWT**	5.2 (3.5-6.5)	5.2 (3.8-6.7)	4 (3.2-6.5)	5.8 (4.8-6.8)
**ΔBWT (mm)**	1.6 (1-3.3)	0.3 (−2.2 to 0.5)	1 (−0.7 to 3.0)	0.4 (−1.5 to 0.5)
**% reduction BWT**	31.8 (26.3-51.2)	7.2 (−54.8 to 9.0)	30.3 (−25.9 to 45.2)	7.5 (−22.8 to 9.7)
**Baseline Modified Limberg score**				
** 3**	0	2		2
** 2**	2	2		1
** 1**	2	1		1
**ΔModified Limberg score**	1 (0.3-1.8)	0 (−1 to 1)	1 (−0.5 to 1.5)	0 (−0.75 to 1.5)
**Baseline MUC**	9.2 (6.9-11.1)	9.3 (7.8-11.3)	7.6 (6.9-11.0)	10.1 (8.7-11.5)
**ΔMUC score**	3.24 (1.8-6.2)	0.42 (−2.6 to 0.6)	3.1 (−0.4 to 5.3)	0.5 (−2.1 to 0.7)

Changes in clinical (SCCAI), laboratory (hemoglobin, albumin, and C-reactive protein), and sonographic parameters from the day of IV cyclosporine initiation to day 3 of cyclosporine treatment. Data are presented as median (interquartile range). Abbreviations: SCCAI, Simple Clinical Colitis Activity Index; MUC, Milan ultrasound criteria = 1.4 × BWT (mm) + 2 × bowel wall flow [present = 1, absent = 0].

Four patients who responded to cyclosporine completed a planned transition to another maintenance therapy: one with vedolizumab, one with ustekinumab, and two with mirikizumab. Of the five patients who did not undergo colectomy during their initial hospitalization, including one patient who switched to upadacitinib during the initial admission, the median follow-up was 3.8 months (IQR 2.7-10.7 months), and none of them needed colectomy or admission by the end of the follow-up period. The median total duration of cyclosporine therapy, including IV followed by oral cyclosporine, was 108 days (IQR 81-135) among responders and 10 days (IQR 6-10) among non-responders.

## Discussion

This is the first study to describe sonographic changes associated with IV cyclosporine in hospitalized patients with ASUC. Our study identified changes in sonographic findings as early as 3 days after treatment initiation with IV cyclosporine in patients with ASUC.

In our patient population, patients who responded to cyclosporine had a 31.8% (IQR 26.3-51.2%) reduction of BWT, while only 7.2% (IQR −54.8 to 9.0) in the non-responder group. Of note, the baseline BWT was similar between the responder and non-responder groups. These findings are consistent with the study by llvemark et al. which showed a > 20% BWT reduction after 48 ± 24 h of intravenous corticosteroids and demonstrated an odds ratio of 22.6 (95% CI, 4.2-201.2) to predict response in ASUC,^17^ although our observations are descriptive and limited to a treatment-refractory cohort who failed multiple advanced therapies and IV corticosteroids.

Assessment of colectomy risk is imperative for the management of ASUC. Piazza et al. reported that the baseline MUC score by IUS, not the Mayo Endoscopic Subscore, was the only independent factor associated with colectomy risk, with an area under the curve of 0.83 (optimal MUC score cut-off of 7.7 in UC).^18^ These findings correspond to our study, in which the baseline MUC score in the patient who underwent colectomy was a median of 10.1 (IQR 8.7-11.5) vs a median of 7.6 (IQR 6.9-11.0) in the non-colectomy group.

In the description between the colectomy versus non-colectomy groups in Table 2 (one patient in the non-responder to IV cyclosporine avoided colectomy after switching to upadacitinib), it is notable that the baseline sonographic findings in the colectomy group were numerically more severe than those in the non-colectomy group (median BWT 5.8 mm with modified Limberg 2.5 vs 4 mm with 2, respectively), while those in the clinical responder to IV cyclosporine group had the same baseline BWT of 5.2 mm as those in the non-clinical responder group. Sonographic findings, including BWT, modified Limberg score, and MUC score, consistently improved on follow-up IUS in both cohorts. These observations suggest that short-interval transmural assessment with IUS may capture early response regardless of baseline sonographic severity, although no causal inferences can be drawn given the descriptive nature of the study.

This case series has several limitations. First, this is a single-center retrospective observational study with a small sample size that limits statistical analysis to adjust for confounding factors such as disease severity. However, the trend of sonographic findings in our study is consistent with previous studies.^17^ Additional limitations of the study are the concomitant IV corticosteroids to the IV cyclosporine therapy given in this cohort of patients and the unavailable IUS data prior to IV corticosteroids. Although this may confound the findings, we believe our described sonographic changes remain important as IV corticosteroids are often concomitantly given with IV cyclosporine therapy. Furthermore, prior to IV cyclosporine initiation, we saw a lack of clinical response; therefore, we believe the observed clinical response to IV cyclosporine is more likely to be attributed to the addition of cyclosporine therapy than to ongoing IV corticosteroid use. Additionally, the sonographers were not blinded to clinical data, and IUS findings were shared with the treating team, which might have influenced sonographic interpretation as well as subsequent decision-making. The absence of standardized criteria for colectomy or initiation of upadacitinib as salvage therapy may have influenced the outcome of colectomy. These decisions reflected real-world practice in the context of emerging evidence for upadacitinib as a salvage therapy and were made at the discretion of the treating attending based on comprehensive clinical evaluation and multidisciplinary discussion. Lastly, the follow-up duration after initiating cyclosporine was relatively short, but our follow-up in all responders was long enough to demonstrate a durable response with subsequent transition to oral maintenance therapy.

## Conclusion

Early sonographic changes were observed as early as 3 days after initiation of IV cyclosporine salvage therapy in patients with ASUC. These findings are observational and hypothesis-generating and warrant further large-scale prospective studies.

## Data Availability

The data from this analysis are available within the article. Any additional data will be shared by the corresponding author upon reasonable request.
